# Short-term action potential memory and electrical restitution: A cellular computational study on the stability of cardiac repolarization under dynamic pacing

**DOI:** 10.1371/journal.pone.0193416

**Published:** 2018-03-01

**Authors:** Massimiliano Zaniboni

**Affiliations:** 1 Department of Chemistry, Life Sciences and Environmental Sustainability - University of Parma Parco Area delle Scienze, Parma, Italy; 2 Center of Excellence for Toxicological Research (CERT) - University of Parma, Parma, Italy; University of Minnesota, UNITED STATES

## Abstract

Electrical restitution (ER) is a major determinant of repolarization stability and, under fast pacing rate, it reveals memory properties of the cardiac action potential (AP), whose dynamics have never been fully elucidated, nor their ionic mechanisms. Previous studies have looked at ER mainly in terms of changes in AP duration (APD) when the preceding diastolic interval (DI) changes and described dynamic conditions where this relationship shows hysteresis which, in turn, has been proposed as a marker of short-term AP memory and repolarization stability. By means of numerical simulations of a non-propagated human ventricular AP, we show here that measuring ER as APD versus the preceding cycle length (CL) provides additional information on repolarization dynamics which is not contained in the companion formulation. We focus particularly on fast pacing rate conditions with a beat-to-beat variable CL, where memory properties emerge from APD vs CL and not from APD vs DI and should thus be stored in APD and not in DI. We provide an ion-currents characterization of such conditions under periodic and random CL variability, and show that the memory stored in APD plays a stabilizing role on AP repolarization under pacing rate perturbations. The gating kinetics of L-type calcium current seems to be the main determinant of this safety mechanism. We also show that, at fast pacing rate and under otherwise identical pacing conditions, a periodically beat-to-beat changing CL is more effective than a random one in stabilizing repolarization. In summary, we propose a novel view of short-term AP memory, differentially stored between systole and diastole, which opens a number of methodological and theoretical implications for the understanding of arrhythmia development.

## Introduction

The longer duration of cardiac action potential (AP), as compared for example with that of skeletal muscle and neuronal cells, has the functional significance of controlling and modulating the pumping cycle of the heart, which, in mammals, takes place in a time scale of some hundreds of milliseconds. Several mechanisms contribute to adjusting the partition of the cycle length into diastole and systole, the mechanical counterparts of the ventricular AP and DI, for the cardiac pump to combine pacing rate with strength of contraction and meet the organism’s energy requirements. More precisely, it is the repolarization phase of AP that chiefly controls the relaxation of the heart chambers, and the kinetics of ion currents involved in this phase determine the rate-dependent adaptation of its duration (APD) [1].

Two main processes govern the rate dependence of APD; the first describes stationary APD changes resulting from changes in cycle length (CL), and is usually referred to as rate dependence (RD) *tout court* [1, 2]; the second, measures APD changes after a sudden test switch from a constant to a variably delayed CL, as a function of the pre-test CL or DI, and is called electrical restitution (ER_CL_ and ER_DI_ respectively) [3, 4, 5].

The normal beating of the heart is endowed with a given amount of beat-to-beat heart rate variability (HRV), which is due both to the ionic mechanism generating pacemaker activity [6, 7] and to a large number of regulatory mechanisms, where the autonomic nervous system plays the leading role [8]. HRV is considered a risk marker in several cardiac pathological states [9], thus the interest in characterizing the complex rate-dependent behavior of ventricular APD in non-stationary pacing conditions by looking at dynamic restitution properties, rather than stationary ones.

A dynamic ER curve (dER) can be drawn by reporting APD versus preceding CL (or preceding DI) [10, 11] for an AP train elicited at variable pacing rate. dRD and dER are also derived from electrocardiographic measurements, where QT, RR, and TQ take the place of APD, CL, and DI respectively. Different meanings have been attributed from time to time to the term “dynamic” when referring to the measure of restitution properties [12, 13, 14], but for the sake of this study we will only refer to the definitions given above. Key features of repolarization dynamics are stability (i.e. the ability to restore a given average AP waveform after a transient perturbation of pacing rate), and short-term memory (i.e. the dependence of an APD on preceding activation properties, such as APDs, DIs, or even ion concentrations) [15, 16, 17]. A widely adopted but still controversial marker of repolarization stability is the ER slope [18, 19], frequently used for example to test the antiarrhythmic potential of pharmacological interventions [20]. The so called “restitution-hypothesis” proposes that tissue with an APD restitution slope > 1 develops APD alternans during rapid pacing. Nevertheless, some evidence has been found that contradicts this hypothesis [19].

A number of different approaches have been proposed to measure short-term AP memory [21–24], while the lack of a unifying explanation mechanism perhaps reflects the need for better comprehension of the phenomenon. A link has been found between memory and stability, since an increase in the former frequently increases the latter [25–28]. Part of the linking mechanism has been associated with the hysteresis behavior assumed by dER during periodically varying fast pacing stimulations [15, 25, 29], where the magnitude of APD at a given DI differs depending on whether the magnitude of DI is increasing or decreasing at that time. One key observation has been made by Wu and Patwardan [25] who showed the effect of hysteresis in dER_DI_ on the evolution of a sudden pacing perturbation, demonstrating that hysteresis tends to buffer activation instability, whereas absence of hysteresis favors APD alternans after the same perturbation.

All previous studies have been done by looking at dER_DI_ [15, 29–31], as opposed to dER_CL_, mainly because of the predictive value attributed to its slope. Hysteresis in dER_CL_ has been shown previously, but with reference to steady-state changes in the type of rhythm (Hopf bifurcations between 1:1, 2:1, 2:2 beating modes) [32, 33], or in the amount and sign of APD response for sudden changes in constant CL values [2, 5]. However, it has never been explored in dynamic conditions and at high pacing rate.

By systematically studying the behavior of a well-established human cardiac ventricular AP model [34] under dynamic pacing conditions, we found that hysteresis in dER_CL_ also develops and carries relevant information on AP dynamics that is not contained in dER_DI_ data, and ought to be taken into account to understand repolarization stability. In doing so, we also found that a given extent of beat-to-beat CL variability, at a high pacing rate, makes ventricular repolarization more stable. In addition to this previously made observation [35], we found that periodic, rather than stochastic, beat-to-beat CL changes are more effective in stabilizing repolarization. What emerges from our study is a novel picture of cardiac AP dynamics under variable pacing, where short-term memory is a rate-dependent property, differentially shared by systole (APD) and diastole (DI) depending on the dynamics of pacing variability.

## Methods

All simulations reported in this study were performed by means of the ten Tusscher et al. 2006 [34] human ventricular AP model, which will be referred to in this manuscript as TP06. The CellML format of the model [36] was recompiled in its Matlab version by means of COR facility at http://www.cor.physiol.ox.ac.uk. The ‘ode15s’ solver built into the R2016a version of Matlab (The Math-Works, Inc., USA) was used to integrate the model equations. All simulations were run on a PC with Intel Core i5, 2.5 GHz CPU. APs were elicited by simulating 3 ms-long current injections with an amplitude 1.5 times the current threshold. AP duration was measured as APD_-60mV_, i.e. the time between the maximum first derivative of membrane potential (V_m_) during the initial fast depolarization phase and the time when V_m_ had fallen to a value of -60 mV. The rate dependence (RD) of APD was measured as follows: at different CLs, from 300 ms up to 1400 ms, step 20 ms, a 1000 beats pacing train was simulated to allow the AP waveform to reach a steady-state configuration. The average values of the last 20 beats of each sequence was taken for each CL and used to draw the RD curve. Classic electrical restitution (ER) was measured by conditioning training the membrane at a given cycle length (CL*), delivering an extra-stimulus delayed to within CL* ± 20 ms, and reporting the resulting APD versus the CL or DI value of the cycle containing the last conditioning beat. ER was also measured during dynamic, random or periodic pacing by saving the 19×1 vector of TP06 variables at the end of the (n-1)^th^ cycle preceding the last conditioning beat (n^th^ beat), and applying the classic ER protocol described above while making the n^th^ CL varying within a given range; thus ER_CL_ is the function f described by APD_n+1_ = f(CL_n_) and ER_DI_ by APD_n+1_ = f(DI_n_). In some simulations the time-dependence of the gating variables for given ion currents (I_CaL_, I_Kr_, and I_Ks_) was removed using the following procedure. A simulation was run at a given CL (after a conditioning train of 1000 beats) and the current-voltage relationship of the ion current of interest flowing during the AP was plotted and fitted with variable order polynomials. The purely voltage-dependent function was then used to update ion current values during AP simulation, instead of solving the corresponding gating equations. The substitution with the voltage-dependent fitted values was not applied within the first 9 ms of the AP potential due to the fast kinetics of currents in that phase and to the fact that our main interest was in late repolarization dynamics (see also the S3 Fig).

## Results

### Rate dependency and electrical restitution

**Fig 1:**
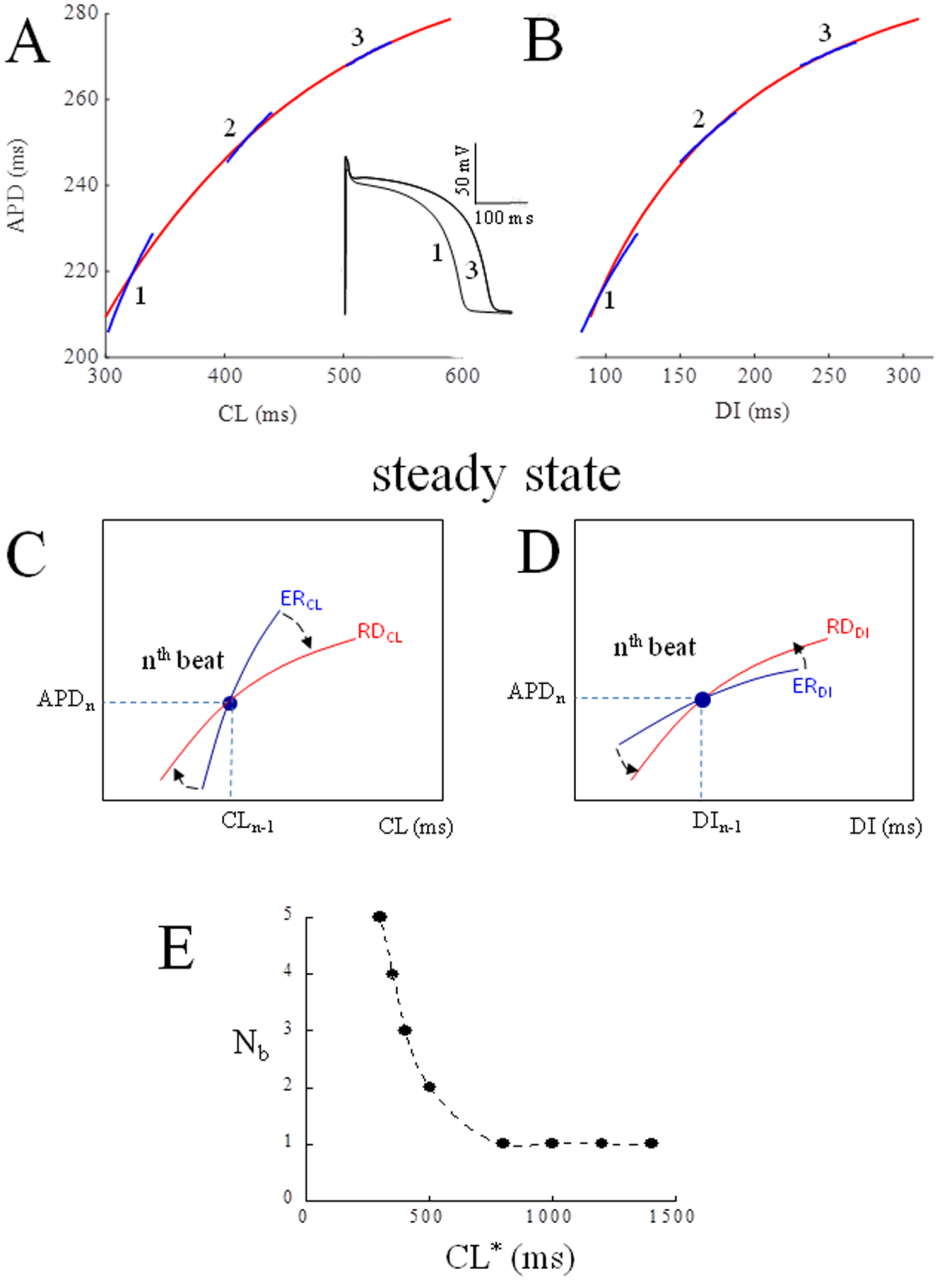
Steady-state. Panel A: Rate dependence (RD) and electrical restitution (ER) of APD (red and blue respectively) were measured after conditioning pacing trains of 1000 beats at constant CL values between 250 and 2000 ms, and shown as APDvsCL. Panel B: same, in the case of APDvsDI representation. Only 3 ER curves are shown in both cases. The inset in panel A shows AP waveforms measured at 320 and 520 ms. Panels C and D show schematically the state of the membrane at the n^th^ beat after a constant pacing train (blue dot), with the angle between ER and RD. The number of beats (N_b_) taken for ER to rotate clockwise (panel C) and anti-clockwise (panel D) into RD is shown in panel E as a function of conditioning CL.

Fig 1A and 1B shows the dependence of the TP06 AP model from pacing rate by reporting RD (red) and ER (blue) curves, the latter derived for 3 values of conditioning CL (320, 420, and 520 ms), measured as ER_CL_ (panel A) and ER_DI_ (panel B). At a given steady-state, if CL suddenly changes to another constant value, APD will instantly change according to ER_CL_ and, after a given number of beats (N_b_), reach a new steady-state given by RD_CL_; in other words, it takes a different N_b_ for each ER curve to rotate (clockwise for ER_CL_ and anti-clockwise for ER_DI_) into the RD curve (panels C and D). N_b_ (and the corresponding time, not shown) decreases mono-exponentially with the conditioning CL (panel E).

### Steady and dynamic AP states

**Fig 2:**
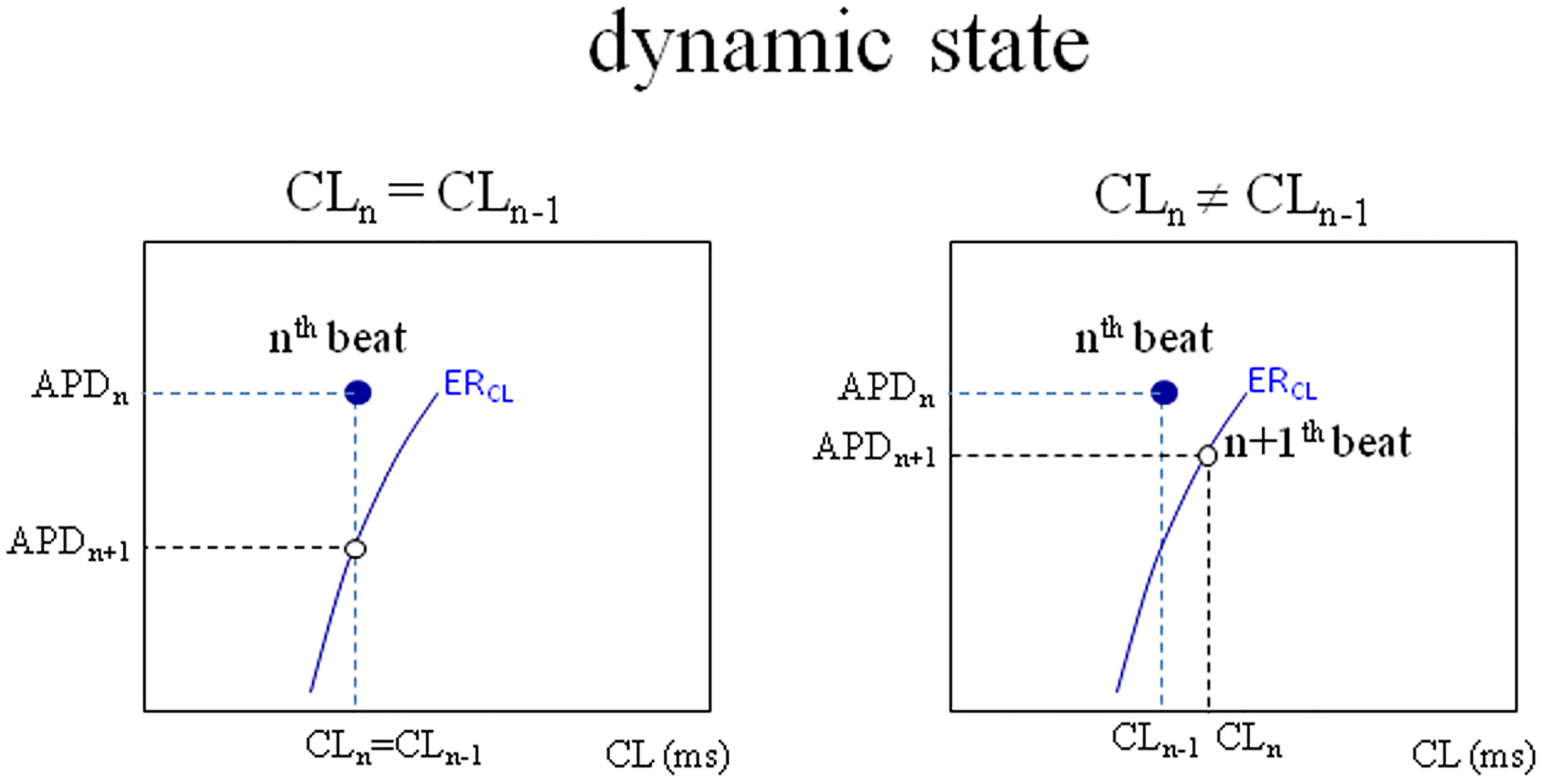
Dynamic state. The scheme represents a classic ER curve measured during a CL-varying pacing protocol in the general case where CL_n-1_ (preceding the last conditioning beat) and CL_n_ (preceding the test beat) are different (right-hand panel), and in the case where they are equal (on the left). Under variable CL pacing, the state of the n^th^ beat (blue dot) does not belong to the ER curve (blue) measured at the same time, which describes instead all the possible states occupied by the n+1^th^ beat.

N_b_ measures the evolution from the sudden (ER) to the stationary (RD) rate dependence of APD for a membrane in its steady-state. Indeed, the membrane characterized in both the RD and the ER protocols described above is in steady-state, i.e. there are no time-dependent electrogenic processes occurring at the time of the measurement. From now on we will call the average value of conditioning CL, “CL*” (consequently, this definition can hold either for constant or variable pacing), and define the state of the membrane for a given n^th^ beat with one point in the (CL, APD)- or (DI, APD)-space with coordinates (CL_n-1_, APD_n_) or (DI_n-1_, APD_n_); all the information needed to assign the state of the n+1^th^ beat is in the ER_CL_ (or ER_DI_) obtained after the n^th^ beat (which we call its ER curve). In steady-state conditions (constant conditioning CL) each state belongs to its ER curve (Fig 1C and 1D); it generally does not do so in dynamic conditions (beat-to-beat variable conditioning CL), regardless of whether CL_n_ is equal or different from CL_n-1_ (Fig 2), whether we are considering ER_CL_ or ER_DI_.

#### Random changes in high pacing rate

An example of dynamic state is that in which the pacing CL changes randomly within a given percentage of a central value. In this case, the time law for CL is:

$$CL(N)={CL}^{*}+clv\;rand(-1,1)$$ (1)

where rand(-1,1) is a random number between -1 and 1, generated for each beat, and *clv* the half range of CL variability. The time at which beats are elicited is:

$$t\left(N\right)=\sum_{i=1}^{N}CL\left(i\right)$$ (2)

**Fig 3:**
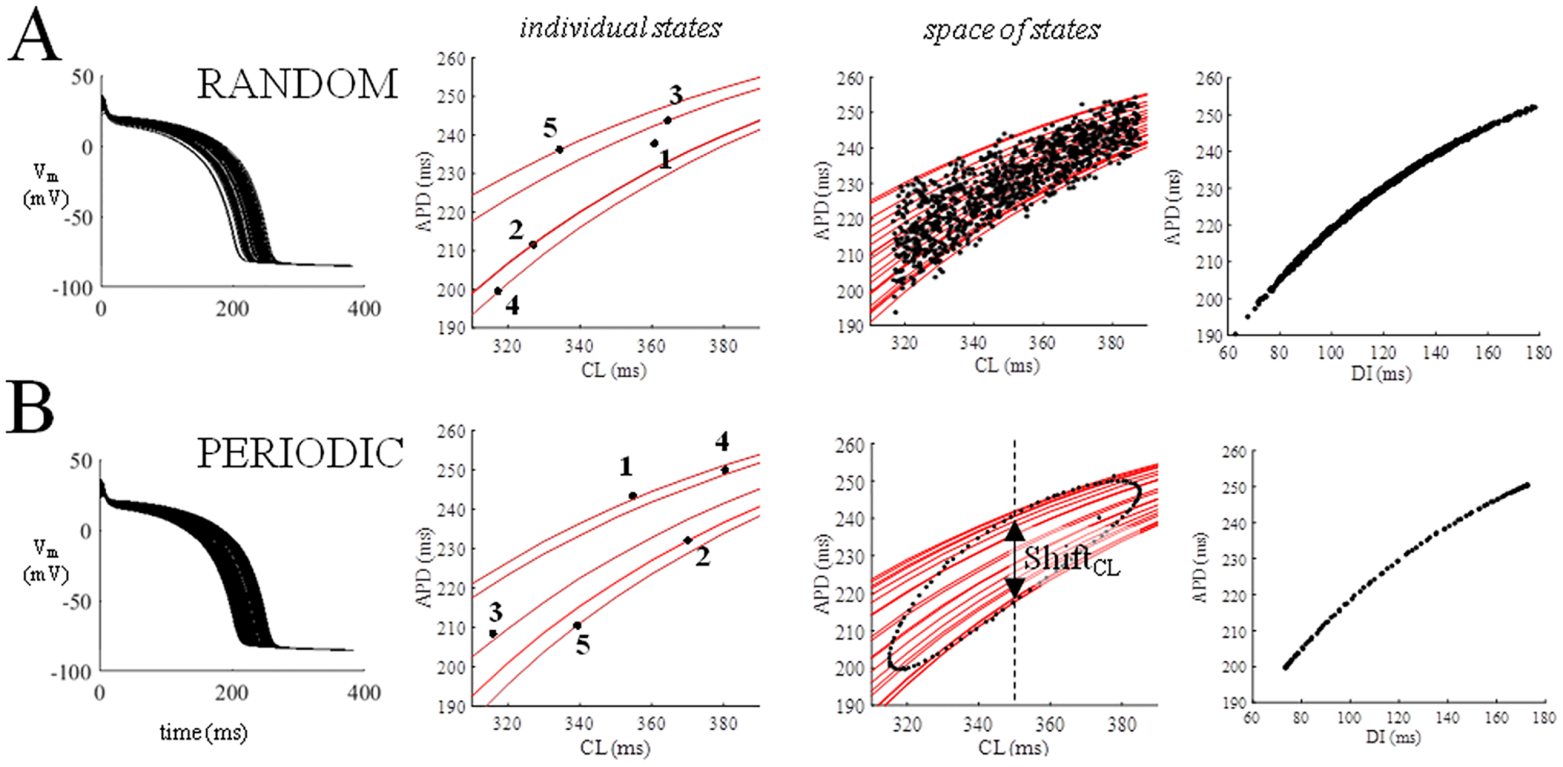
Random and periodic pacing. Twenty consecutive APs simulated under random and periodic pacing (CL* = 350 ms, clv = 35 ms, ɷ = 2.4) are shown in the left-hand column. Five consecutive states and corresponding classic ER_CL_ curves (in red) are shown in second column for each sequence. The collection of states over 1000 beats (random) and 200 beats (periodic) sequences are shown on the third column, superimposed on the corresponding family of ER_CL_ curves. Shift_CL_ (double arrow, bottom third panel) is defined here as the vertical width of the space of states at CL*. The fourth column shows the space of states as APD vs DI representations in both instances.

Twenty consecutive superimposed APs (from a sequence of 1000) simulated under random pacing (CL* = 350 ms, *clv* = 35 ms) are shown in the left-hand panel of Fig 3A. There is no fixed relationship between consecutive CLs, since they can assume any value within CL*± 35 ms. Given that the states do not belong to their ER curves, the system moves beat-to-beat on a family of ER curves (Fig 3A, second column) along a random path. We will call the collection of the states occupied by the system the “space of states”. The space of states for random pacing takes a trapezoidal shape (third column), limited superiorly by the highest ER curve of the family and inferiorly by the lowest one. We note that, for the same conditions, the space of states in the APD vs DI representation is a single ER_DI_ function (fourth column). Moreover, the vertical width of the family of ER_CL_ curves decreases in parallel with the pacing rate and, for CL > 500 ms, the space of states essentially coincides with a single ER_CL_ curve.

#### Periodic changes in high pacing rate

Another example of a dynamic state is that in which the pacing CL oscillates within a given range around the central CL* value. This can be simulated by making CL change like:

$$CL(N)={CL}^{*}+clv\;\text{sin}\left(\omega \;N\right)$$ (3)

where ɷ is the frequency of oscillation. Thus, the time at which beats are elicited is:

$$t\left(N\right)=\sum_{i=1}^{N}CL\left(i\right)={\text{N}\;\text{CL}}^{*}+clv\;\sum_{i=1}^{N}\;\text{sin}\left(\omega \;i\right)$$ (4)

and 2π/ɷ is the number of beats required for a complete oscillation. A sequence of consecutive APs simulated according to Eq 3 (CL* = 350 ms, *clv* = 35 ms, ɷ = 2.4 4) is shown in Fig 3B. Consecutive beats belong to a family of ER_CL_ curves, although in this case their path is forced by the regular oscillations of CL to move clockwise along a closed hysteresis loop, which represents the space of states for this type of pacing variability. Again, for the same conditions the space of states in the APD vs DI representation is a single ER_DI_ function (fourth column). In addition, as pacing frequency decreases, for the same pacing parameters, the hysteresis loop collapses into a single ER_CL_ curve. Spaces of states under beat-to-beat CL-varying pacing describe what we have called dynamic ER (see Methods section), which we will refer to as dER.

A third type of dynamic state is that in which the pacing CL alternates around the central value CL*, such as CL(N) = CL* + (-1)^N^ *clv* [37]. In this case, consecutive beats belong to only two ER_CL_ curves, which are the upper and the lower ones of the family of ER_CL_ curves obtained by the pacing protocol described by Eqs (1) and (3). Results from this alternating protocol were reported at the 42^nd^ EWGCCE Meeting of the European Society of Cardiology [38], but have not been included in the present study.

### Short-term memory

The three pacing protocols described above reveal an intrinsic property of AP repolarization that is associated with short-term AP memory via the transition from a single to a family of ER curves, and only emerges in certain conditions. Thus, we quantify AP memory as the vertical width of the space of states, i.e. the distance between the lower and upper ER curves, which we will call, depending on the type of representation adopted, Shift_CL_ and Shift_DI_, since they measure the vertical shift of the family of ER curves introduced during dynamic pacing (see Fig 3B). A necessary condition for such a shift to develop is a high pacing frequency, and a key factor is the size of the beat-to-beat CL changes which, under random pacing is uniquely determined by *clv*, whereas under periodic pacing it is determined by *clv* and ω.

**Fig 4:**
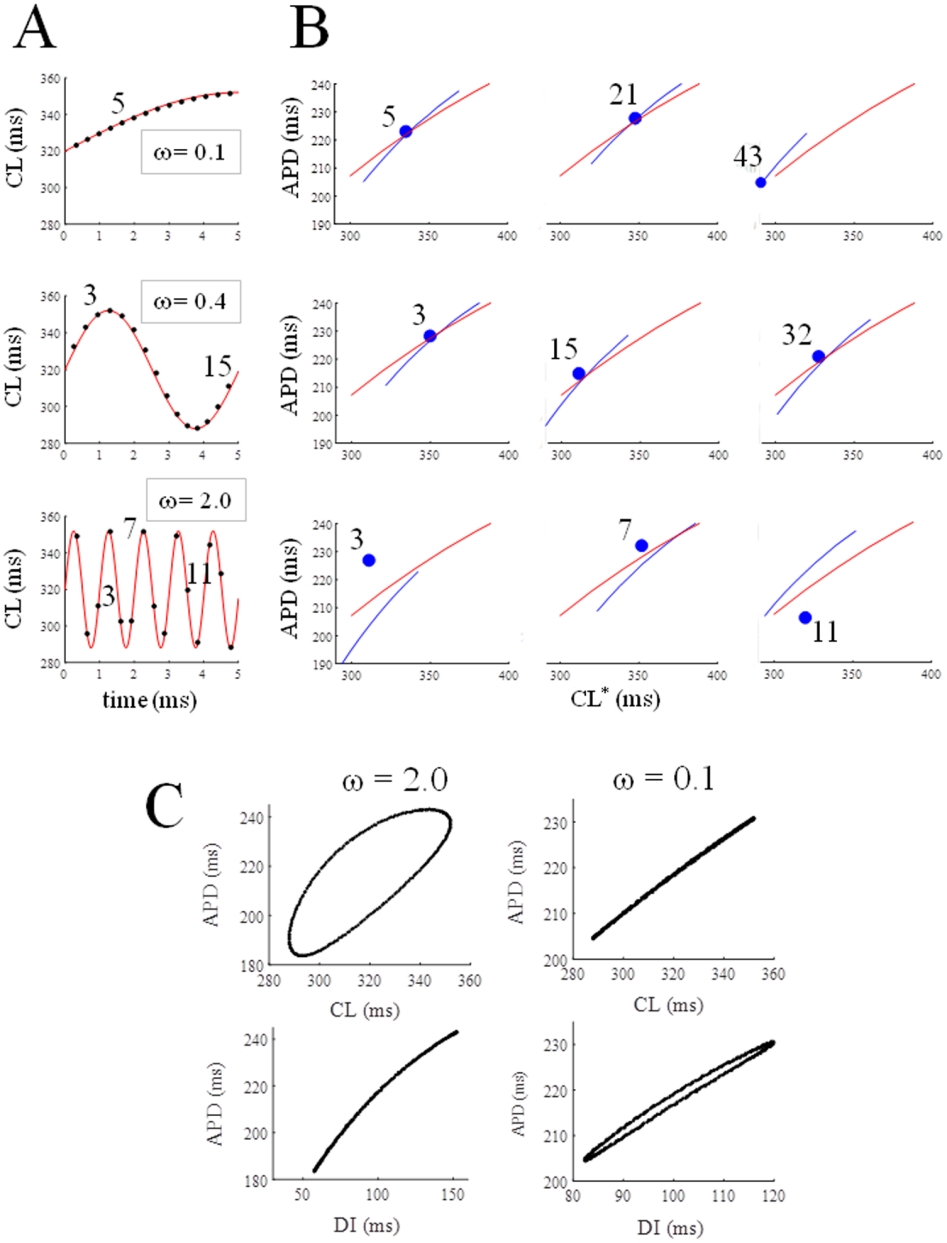
Periodic pacing. (A) Trains of APs were simulated with CL changing periodically (CL* = 320 ms, clv = 32 ms) on a beat-to-beat basis according to Eq 3 and with different values for angular frequency ɷ. (B) The states of 3 beats of each sequence are shown as blue dots. After each of these beats, classical ER_CL_ curves were measured and shown in blue, together with RD (red). (C) Space of sates as dER_CL_ and dER_DI_ for high and low ɷ values.

Fig 4A shows the time course (according to Eqs 3 and 4) of CL for simulated AP trains elicited at 3 different ɷ values (same CL* = 320 ms, same *clv* = 32 ms). Three beats for each sequence are shown in panel B with their state and ER curve (blue dot and blue curve), together with the RD curve (red). For small ɷ values, the states, as in the case of steady-state, belong to their ER curves, at the intersection with the RD curve (panel B, top). As ɷ increases, the average (through consecutive beats) distance between states and ER curves increases. Shift_CL_ is negligible when ɷ is low (< 0.2) but becomes significant for higher ɷ values, leading to hysteresis (top panels in Fig 4C). The opposite is true for ER_DI_ (bottom panels in Fig 4C), where hysteresis is absent for high ɷ values and start to appear for low ɷ values.

**Fig 5:**
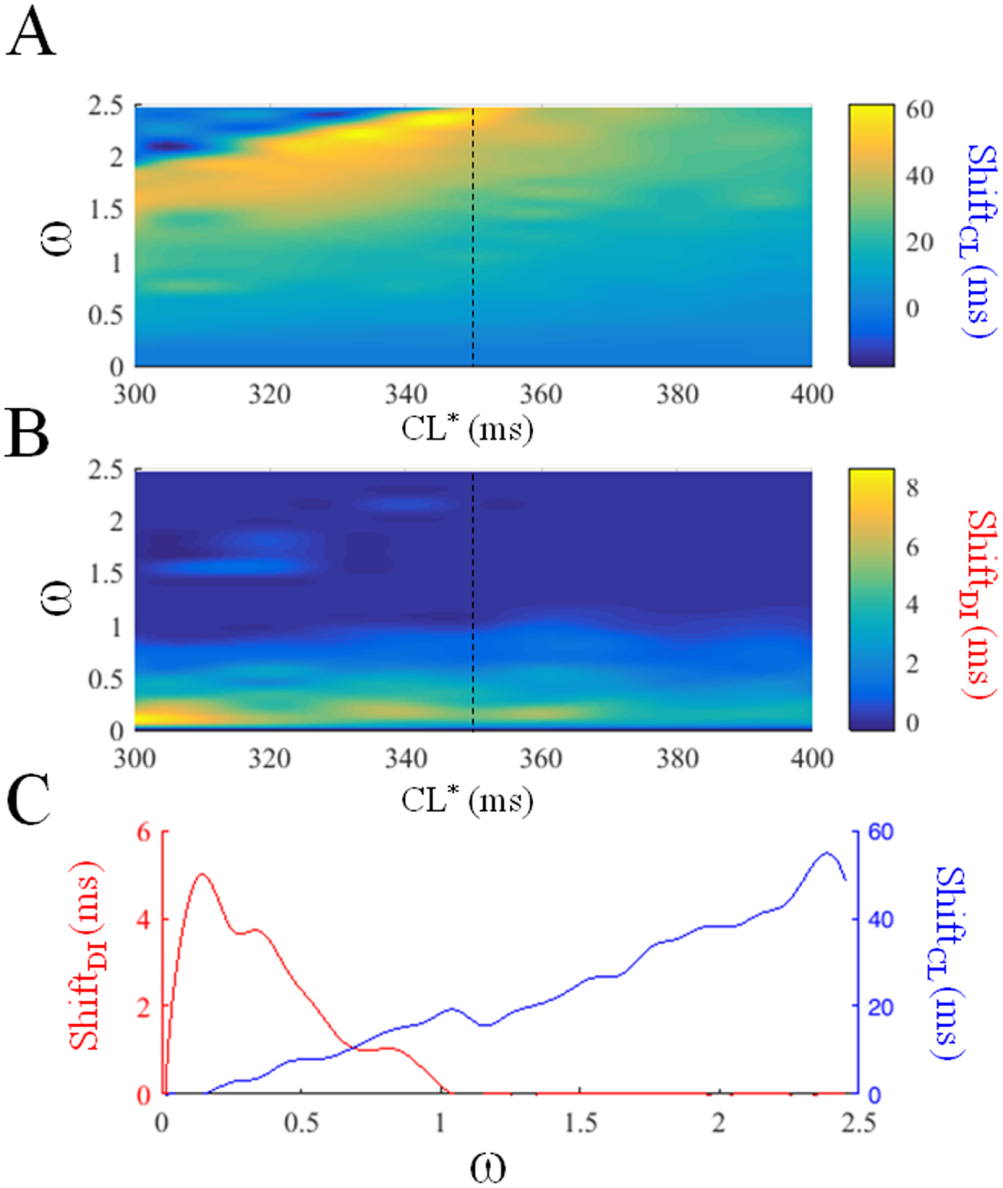
Shift_CL_ and Shift_DI_. dER_CL_ and dER_DI_ were measured as in Fig 4 under periodic pacing for ɷ varying from 0.1 to 2.5 step 0.05 and for CL* varying from 300 ms to 400 ms step 10 ms, and shown in the top and middle panels respectively; the value of Shift_CL_ (A) and Shift_DI_ (B) are shown along the z-axis in color code (note the different scales). Panel C shows Shift_CL_ and Shift_DI_ versus ɷ for CL* = 350 ms (vertical broken line in color panels). The lack of smoothness in Shift_CL_ and Shift_DI_ along the ɷ and CL* directions is due to the numerical resolution of the corresponding simulations described above.

The results of a closer inspection for hysteresis in dER_CL_ and dER_DI_ under periodically changing pacing and for different values of CL* (300 to 400 ms, step 10 ms, *clv* = 70 ms) and ɷ (0.0 to 2.5 step 0.1) are shown in a color code (note the different scale) in Fig 5. The dependence of both Shift_CL_ and Shift_DI_ from ɷ for a CL* = 350 ms (see also vertical broken line in panel A and B) is shown in panel C. When pacing CL varies periodically within ± 70 ms around a value of 350 ms, hysteresis in dER_DI_ appears only within a narrow range of small ɷ values, whereas dER_CL_ hysteresis is present and much larger for most of the ɷ values and increases with ɷ. Thus, hysteresis in dER develops at high pacing rate under periodically varying CL, where this memory effect is shared between APDvsCL and APDvsDI representations, depending on the rate of beat-to-beat CL changes.

**Fig 6:**
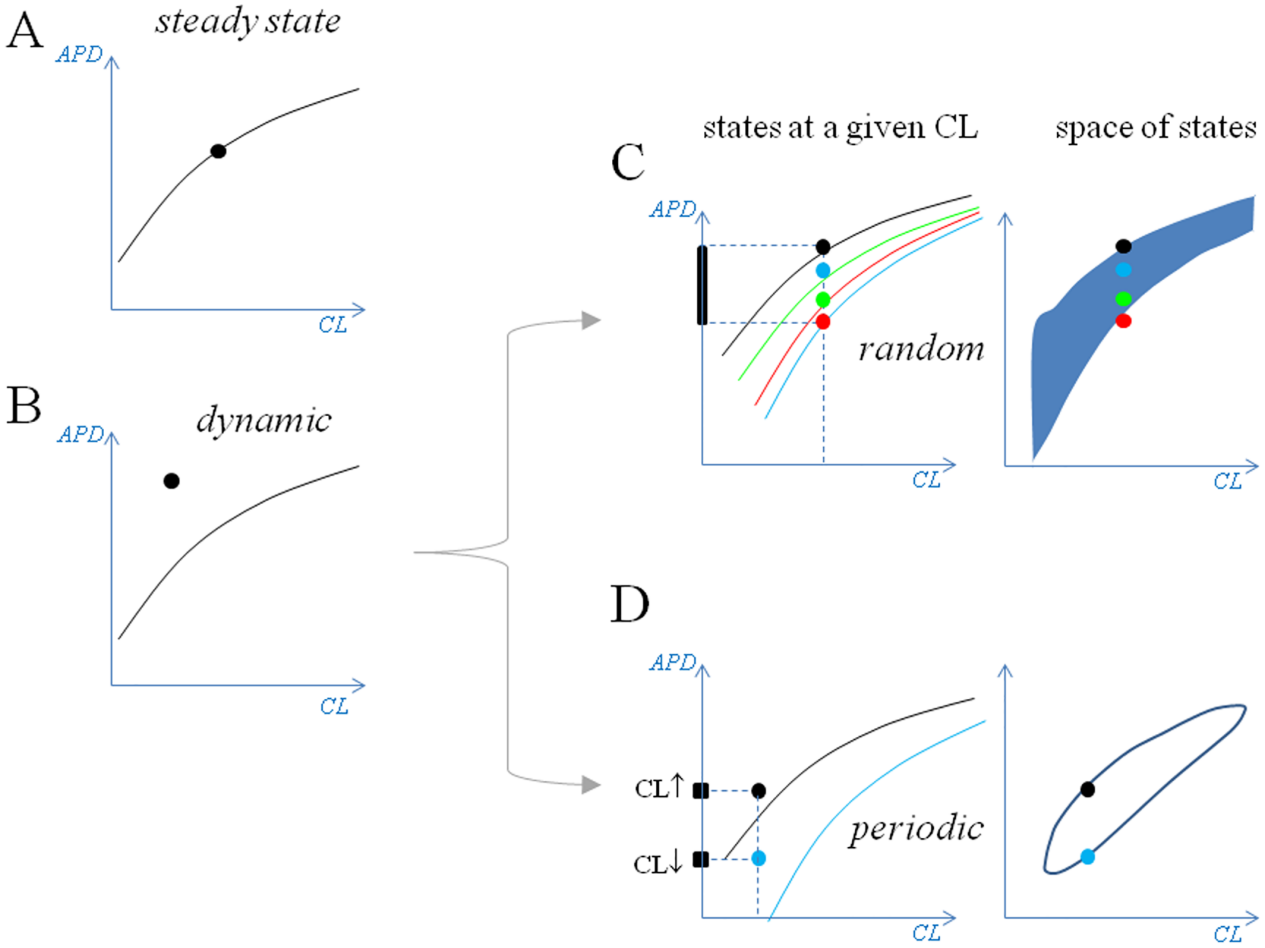
States and space of states. The steady-state of the paced membrane can be described as a CL,APD point lying on its ER curve (A), whereas in dynamic pacing conditions the state does not belong to its ER curve (B) in general. (C) If pacing CL changes randomly, for each CL (vertical broken line) the system can assume an infinite number of states between the lower and upper ER curves of the family (red and black dots respectively); the space of allowed states fills the entire blue area on the right-hand panel. (D) If pacing CL changes periodically, for each CL the system can assume only 2 states, depending on whether CL is increasing (CL↑) or decreasing (CL↓), leading to a closed curve as a space of states (right-hand panel).

A summary of the dER configurations associated with constant and dynamic pacing is schematically shown in Fig 6 in the case of dER_CL_ representations, and can be extended to dER_DI_. Three additional AP ventricular models, the human Priebe and Beuckelmann [39], the O’Hara and Rudy [40] and the rabbit Mahajan et al [41], were tested for the same properties and showed qualitatively similar results, the main difference being related to the CL range (at high pacing rate) where the hysteresis effect appears. This range was always chosen to avoid the CL range for period-doubling bifurcation where APD alternans occurs, when constant pacing CL is lowered below 210 ms (O’Hara and Rudy model), 345 ms (Priebe and Beuckelmann), 239 ms (Mahajan model), and 250 ms (TP06 model). The same range was recently explored in a canine model of ventricular AP by McIntyre and colleagues [42] who found that beat-to-beat CL variability (0–6%, rather than the 10–20% considered here), promotes alternans by shifting the upper limit of its occurrence towards higher values.

### Ion currents and dER during periodic pacing

**Fig 7:**
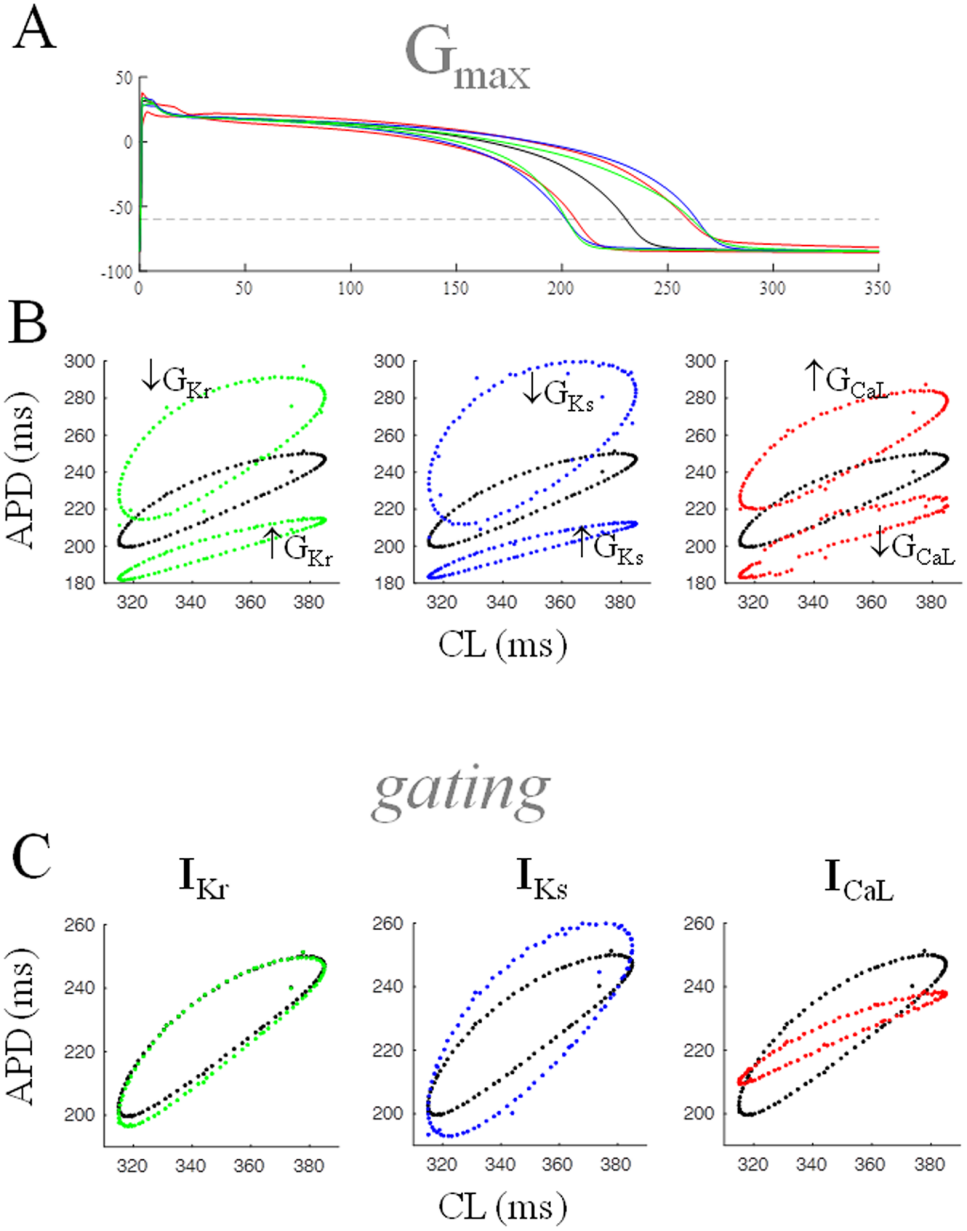
Role of voltage and time-dependence of ion currents. (A) TP06 AP waveform under constant pacing at CL = 350 ms is shown (black), together with modified waveforms obtained by decreasing or increasing G_Kr_, G_Ks_, and G_CaL_ (green, blue, and red respectively) in order to obtain ±12.5% APD changes. (B) Control and modified (as in A) AP waveforms underwent periodic pacing with CL* = 350 ms, clv = 35 ms, and ɷ = 2.4, and the corresponding space of dER_CL_ states are shown in black and in color respectively. (C) Removal of time-dependency did not produce changes in the space of dER_CL_ states in the case of I_Kr_, almost doubled the size of space of dER_CL_ states in the case of I_Ks_, and nearly abolished it in the case of I_CaL_.

So far, we have shown that the space of AP states is one point at constant-, a closed curve in periodic-, and an entire surface in random pacing-conditions (Fig 6). Here we analyze the three ion currents mainly responsible for AP repolarization in the human ventricle because of their role in determining and modulating the size and shape of the space of states under periodic pacing (Fig 7).

#### Role of G_max_

The steady-state APD of the TP06 model was prolonged by the same amount (12.5%) by decreasing, in turn, G_max_ of I_Kr_ or I_Ks_, and increasing G_max_ of I_CaL_ (-90%, -60%, and +90% respectively). An opposite maneuver (+100%, +98%, and -40%) led to a 12.5% decrease in APD in the three cases (Fig 7A). The dER_CL_ was then measured by simulating a pacing train with a CL varying periodically according to Eq 3 (ɷ = 2.4, CL* = 350 ms, *clv* = 35 ms), first in control conditions (black), and then with modified G_max_ values. The greatest increase in the hysteresis loop was obtained under a decrease in G_Ks_ (blue). Results obtained with randomly varying CLs led to comparable results where spaces of states were trapezoidal surfaces instead of a closed loop (not shown). When, from the same data, dER_DI_ curves were derived for the same pacing conditions, they resulted as single unimodal curves in all instances; the decrease in G_Kr_ led to an increase in the dER_DI_ slope from 0.49 (control) to 0.71, a decrease in G_Ks_ to 0.83, and an increase in G_CaL_ to 0.62.

#### Role of time-dependency

A time-independent version of each of the three ion currents was obtained by recording the current during a simulated AP in steady-state control conditions (CL = 350 ms), fitting its current-voltage relationship with a polynomial, and replacing the corresponding TP06 gating equations with the purely voltage-dependent fit. The TP06 model was then paced as in the previous section and dER_CL_ measured in control (Fig 7C, black), and in conditions where, in turn, I_Kr_, I_Ks_, and I_CaL_ were purely voltage-dependent during AP repolarization phase (green, blue, and red respectively). Whereas the time-dependence of I_Kr_ does not seem to play a role in dER_CL_ hysteresis, the removal of the time-dependence of I_Ks_ increased Shift_CL_ while that of I_CaL_ almost abolished it under periodic pacing. The time-course of the three ion currents after the fitting procedure can be seen in the S3 Fig.

### Perturbing AP states

Monitoring dER under different pacing conditions helps to study AP repolarization dynamics when a given relatively regular pacing is suddenly perturbed and immediately restored. Perturbation may be in the form of a pre- or post-mature stimulus or, as in our case, of a single missing beat, which, when seen from the ventricle, can be thought of as a pause in the SA nodal firing or as a single failure in AV nodal conduction.

**Fig 8:**
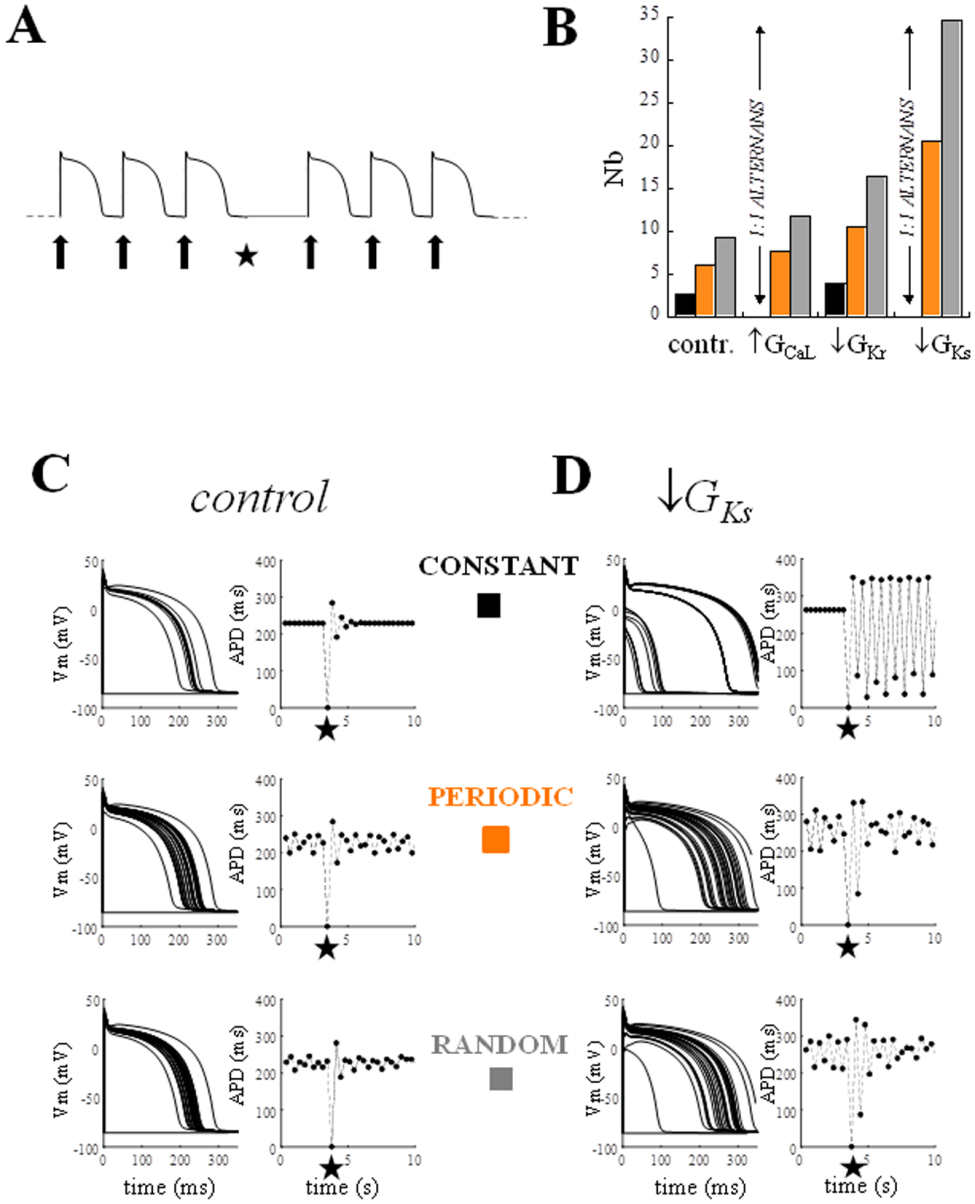
Variable pacing and stability after a missing beat. AP trains under different pacing conditions were perturbed by turning off the current stimulus (star) for one beat (A). The difference between APDs following the missing beat and those of the unperturbed sequence were measured and the number of beats (Nb) required for such a difference to reach a value < 2 ms measured. (B) The histogram shows Nb measured during constant (black), periodic (orange), and random (grey) pacing (CL* = 350 ms, clv = 35 ms, ω = 2.4) in control conditions and for each AP prolonging treatment (see Fig 7). In the case of an increase in G_CaL_ and a decrease in G_Ks_ the missing beat triggered stable APD alternans. A transient alternans between short APD values was also present under a decrease in G_Ks_, but progressively disappeared within 30 beats. (C) Example of data generating histogram in B. Consecutive AP waveforms (left-hand column) and APD time course (on the right) are shown for 10 s simulations in control conditions under constant, periodic, and random pacing and with a missing beat (star) in each sequence. (D) Same for AP prolonged by decrease in G_Ks_.

Fig 8 shows results obtained by simulating pacing trains where CL was, in turn, kept constant, made to change periodically (ɷ = 2.4, *clv* = 35 ms), or randomly (*clv* = 35 ms). At the 10^th^ beat of each sequence the current stimulus was set to zero (star), so that no AP was elicited, and immediately restored in the following cycle (panel A). The number of beats (N_b_) required for each AP train to resume the un-perturbed APD sequence (with a ΔAPD < 2 ms) after the pause was measured for the 3 protocols.

For the sake of the internal clarity of this discussion, and without claiming generality on this delicate theoretical issue, we use the term ‘stability’ here as the ability of repolarization to resume its unperturbed state after a pacing perturbation, which, in the present setting, takes the form of a missing beat. Beat-to-beat variable pacing is always associated with greater Nb (smaller stability) than constant pacing (Fig 8B), even if Nb value is progressively decreasing (and stability increasing) with the rate and amplitude (ɷ and *clv*) of changes and is always smaller under periodic than random ones (see S1 and S2 Figs).

**Fig 9:**
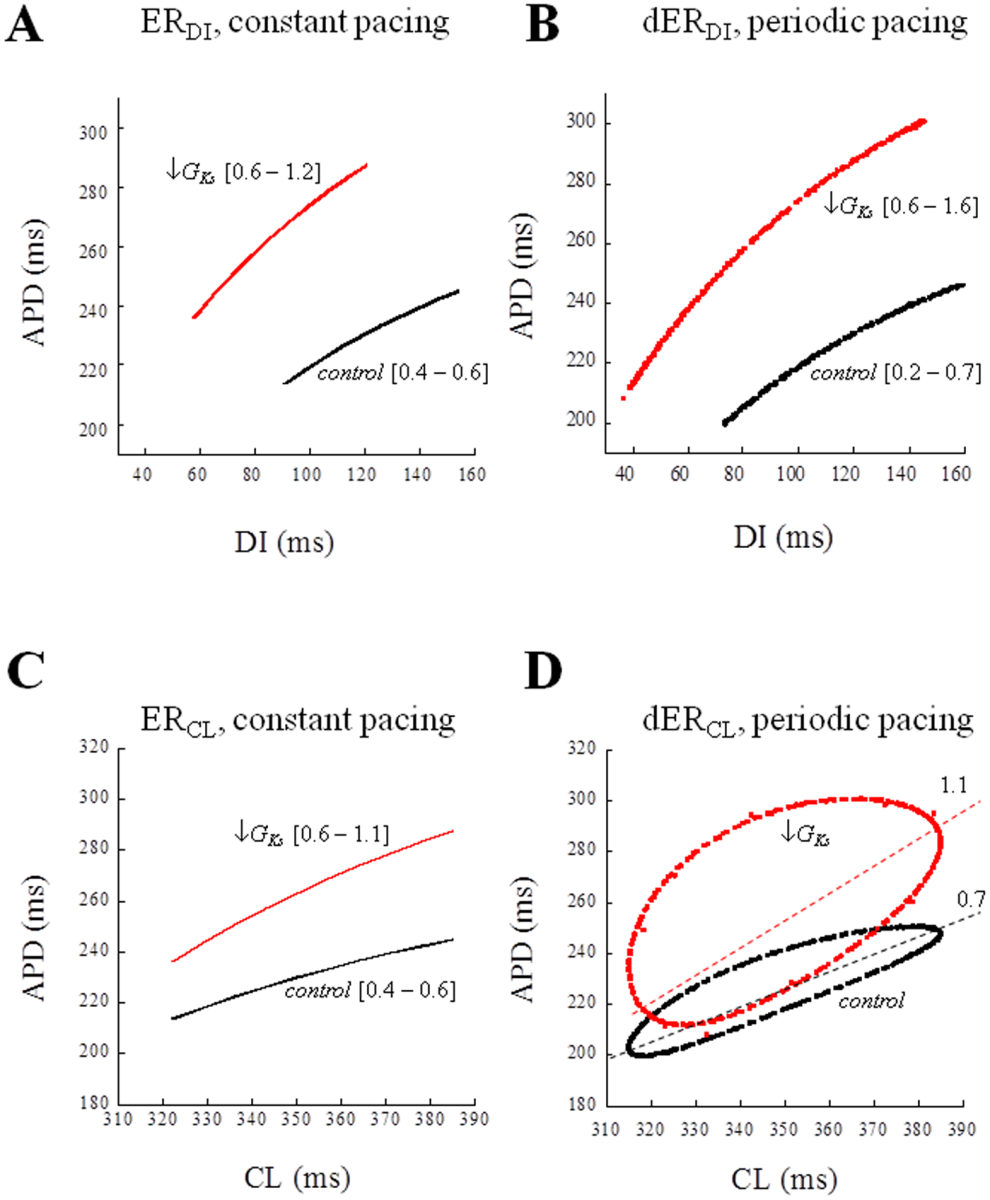
ER during constant and dER during periodic pacing. Classic ER_DI_ (panel A) and ER_CL_ (panel C) and dynamic dER_DI_ (panel B) and dER_CL_ (panel D) were measured before the perturbation (missing beat) in simulations, constant and periodic, like those in Fig 8C and 8D, in control conditions (black) and in diminished G_Ks_ conditions. In brackets, the range of slopes when CL was made to change ±35 ms around 350 ms. In the case of dER_CL_ (panel D) the slope of hysteresis was approximated by measuring the slope of the major axis (dotted lines).

Since N_b_ can change slightly in dynamic control conditions, depending on the position of the missing beat within the sequence, we will consider its average over several beats here; it increases from 2.6 during constant pacing, to about 6.0 during periodic pacing, and 9.2 during random pacing (Fig 8B). When APD was prolonged by increasing G_CaL_ or decreasing G_Ks_, a stable 1:1 alternant APD oscillation (within the simulated 500 beats) arose each time a beat was missing during constant pacing at CL* = 350 ms, whereas APD prolongation obtained by decreasing G_Kr_ only led to an increased N_b_ (with respect to the control). Consecutive AP waveforms and the time course of corresponding APDs are shown in Fig 8 for the three protocols under control conditions (C), and in the case when APD was prolonged by decreasing G_Ks_ (D). Classic ER_DI_ and ER_CL_ curves measured under constant pacing as described in Methods, are shown in Fig 9A and 9C for the two conditions, control (black), and diminished G_Ks_ (red), adopted in Fig 8C and 8D. The numbers in square brackets show the range of the ER_DI_ and ER_CL_ slopes when, as in the present case, the CL of the restitution protocol was made to change within ±35 ms around CL* = 350 ms. When measured at the constant conditioning value of CL (350 ms) or DI (119.1 ms in control, and 85.9 ms under treatment), the 60% decrease in G_Ks_ led to an increase of the ER_DI_ and ER_CL_ slopes from 0.5 to 0.8. In the same figure the dER_DI_ and dER_CL_ representations of periodic pacing simulated in Fig 8C and 8D are also shown in panels B and D respectively. The dER_DI_ slope reaches values up to 1.6 under G_Ks_ decrease, whereas the slope of the major axis of the hysteresis loop of dER_CL_ goes from 0.7 to 1.1 in the same conditions.

**Fig 10:**
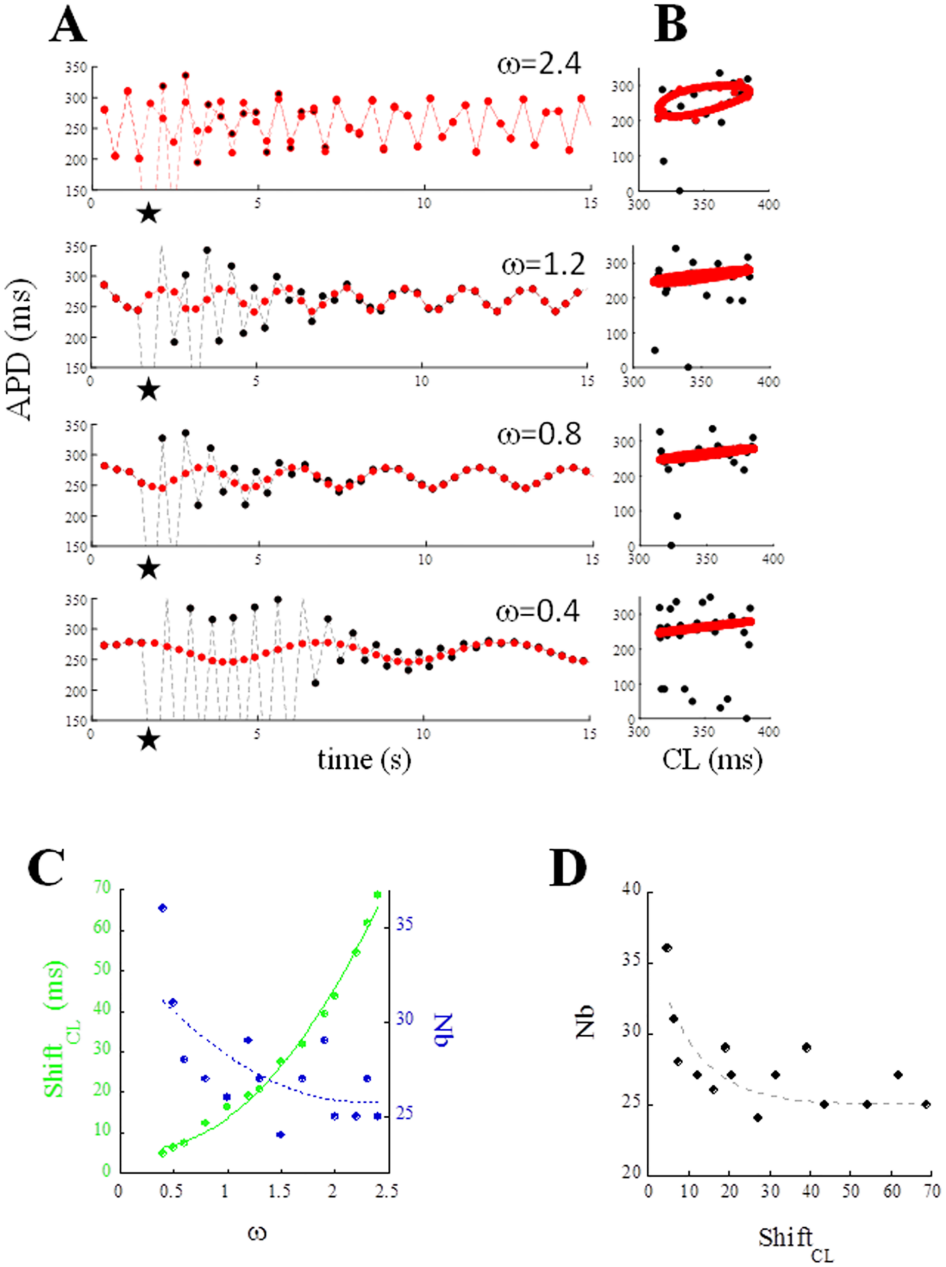
Stabilizing effect of ω. At the same CL* = 350 ms and for the same clv = 35 ms, CL was made to change periodically (Eq 3) with different values of angular velocity ω. (A) Time course of APD over 15 s of periodic pacing was simulated twice for each of the 4 ω values: a first time as control (red), and a second time when the 4^th^ beat of the sequence was missing (black). (B) Same sequences are shown here as space of dER_CL_ states. (C) Number of beats (Nb, blue) required, for each ω, in order for APD to recover the unperturbed value. The size of each hysteresis dER_CL_ loop (Shift_CL_) is also shown in green. Both fitted with mono-exponentials. (D) Same data of panel C, also fitted with mono-exponential.

For a given *clv* value, repolarization stability depends on the frequency of CL changes (ω). This is shown in Fig 10, where the TP06 model, APD-prolonged by a 60%-decrease of G_Ks_ and paced with a constant CL* = 350 ms, always developed APD alternans after a missing beat (star). When paced with a CL periodically varying around 350 ms (*clv* = 35 ms) and with a high enough angular frequency (ω = 2.4), dER_CL_ developed hysteresis, and alternans was stopped after only a few beats (top panels A and B). As ω decreases, the vertical width of the hysteresis loop (Shift_CL_) decreases as well (Fig 10C, green), while the number Nb of post-perturbation beats falling out of the hysteresis loop increases (Fig 10C, blue). It should be noted that, the anti-arrhythmic effect (ability of preventing alternans) obtained for ω > 2.0 is achieved despite the expected pro-arrhythmic increase in the dER_DI_ slope (single unimodal curve at this ω values) up to values greater than 1 (Fig 9B). Thus, if on the one hand pacing variability within a given range (clv) tends, per se, to make repolarization less stable (Fig 8B), on the other the rate of beat-to-beat CL changes (ω and, with it, Shift_CL_) counteracts this effect. This is particularly evident under G_Ks_ downregulation, when a single missing beat during fast pacing leads to stable APD alternans, which is prevented by large beat-to-beat oscillations of CL (Fig 8).

**Fig 11:**
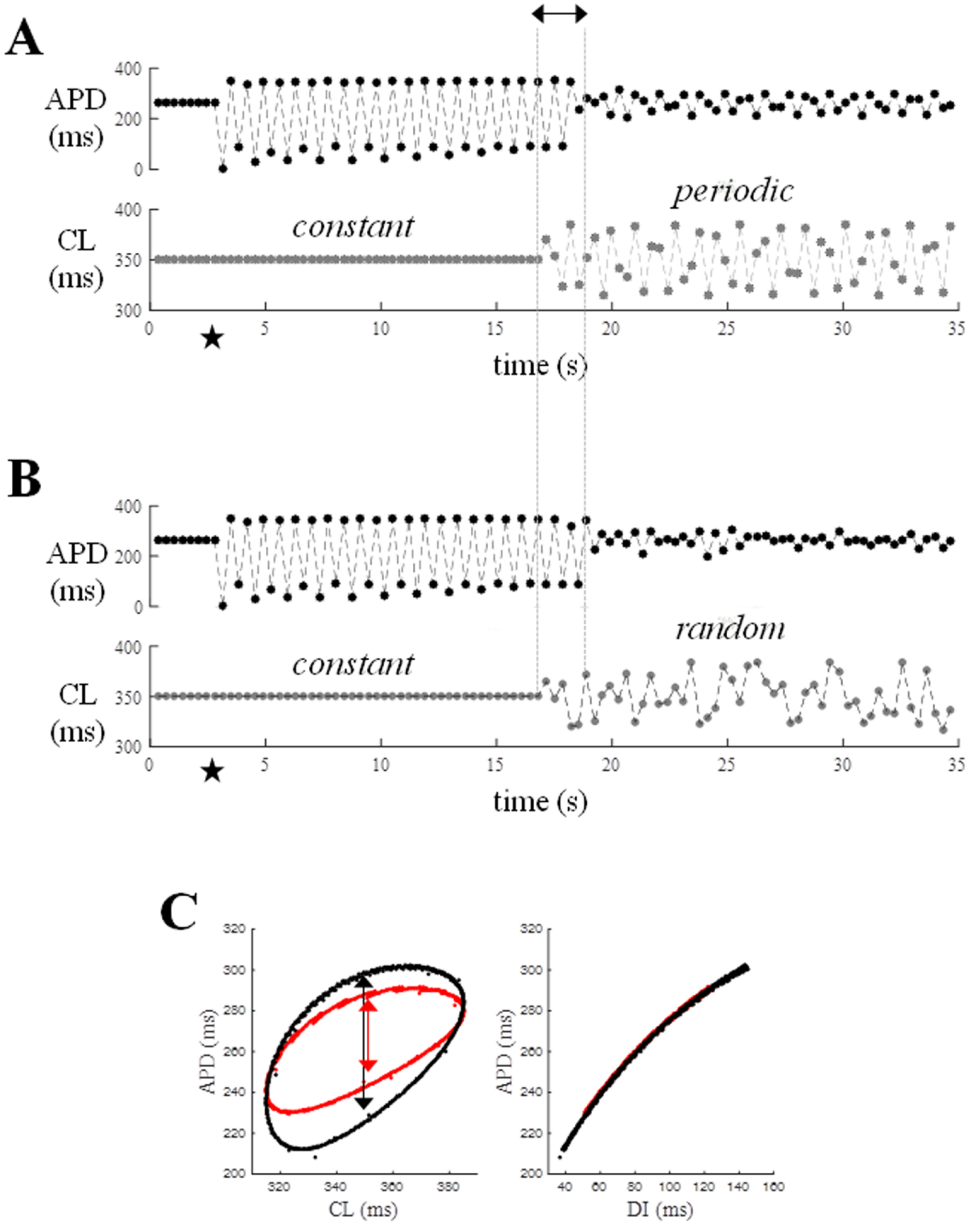
Switch from constant to dynamic pacing. A pacing protocol on the modified TP06 AP model (APD prolonged by decreasing G_Ks_) was simulated where CL was initially kept constant (350 ms) for 17 s and then made to change periodically according to Eq 3 (ɷ = 2.4, clv = 35 ms) (lower panel A). Corresponding APDs following each CL are shown in the upper panel. Stimulus was turned off at the 10^th^ beat of the sequence (star). The missing beat triggered APD alternans during constant pacing, (top panels A and B) which was suddenly quenched after switching to periodic pacing. Same protocol in the case of switching from constant to random pacing (same CL and clv) is shown in panel B. Panel C shows space of dER_CL_ (left) and dER_DI_ (right) states, as measured during periodic pacing (ɷ = 2.4) in panel A (black) and, in identical conditions with ɷ = 2.0 (red).

Not only can a randomly or periodically changing pacing frequency prevent persistent APD alternans, which develops at a constant high pacing rate; it can also stop it when already established, bringing the system back to its unperturbed space of states. This is shown in the simulations in Fig 11: the AP model was first paced for about 17 s at a constant CL* = 350 ms, which was suddenly switched to a periodically (ω = 2.4, *clv* = 35 ms) or randomly (*clv* = 35 ms) varying CL sequence. A single missing beat (star) during constant pacing made APD alternate. Alternans was stopped within a few beats (double arrow) after the switching. If beat-to-beat periodic CL changes were slowed (ω≤ 2.0), the switch to variable pacing did not stop the alternans. A comparison of the space of dER_CL_ and dER_DI_ states for periodic pacing at ω = 2.4 and ω = 2.0 is reported in Fig 11C; whereas Shift_CL_ decreases from 73 ms to 44 ms (double arrows in figure), the shape and the slope of dER_DI_ remain unmodified.

**Fig 12:**
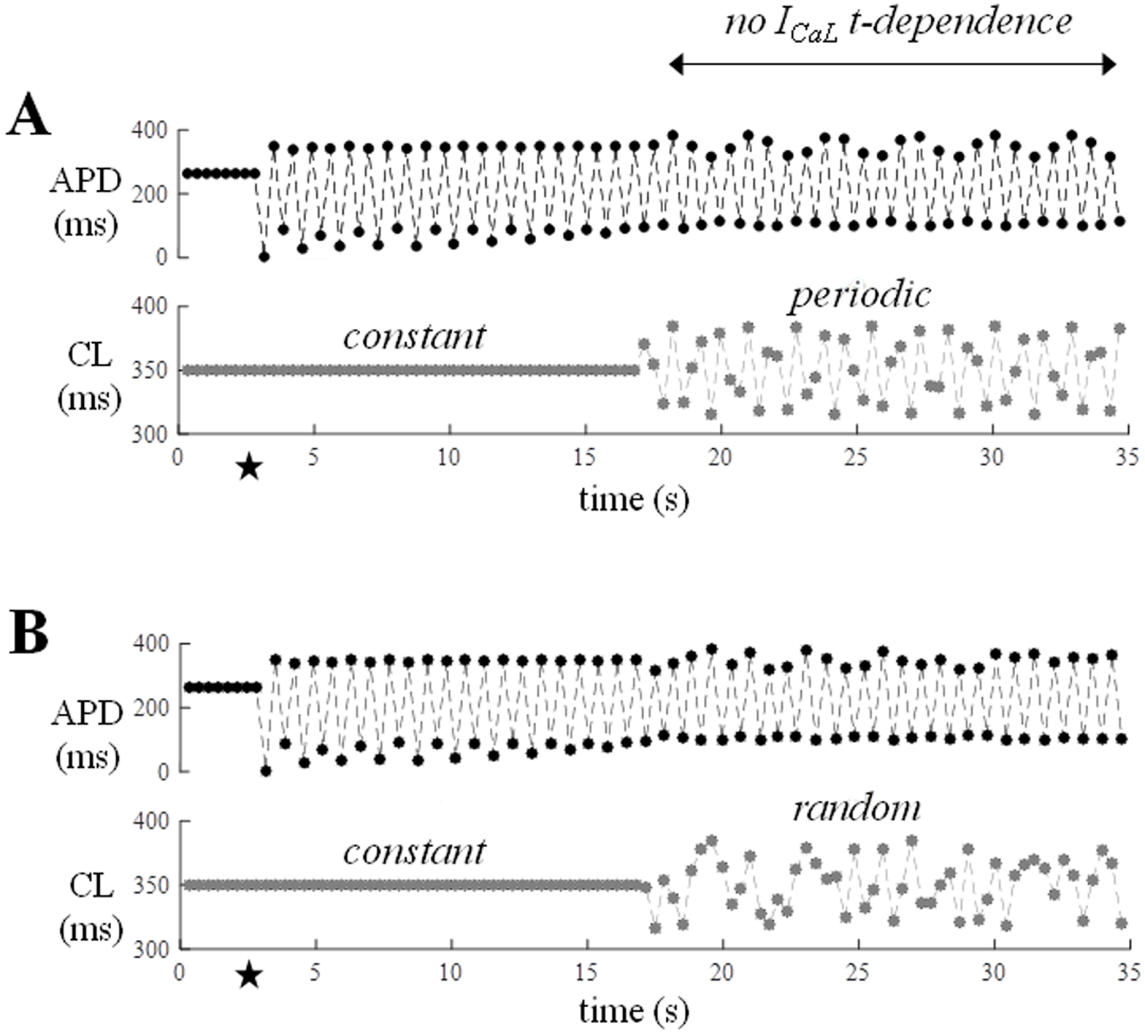
Removing time-dependence of I_CaL_. Same simulations as Fig 11 are shown where, in addition, at the time of switching to variable pacing, the time-dependence of I_CaL_ was turned off from the TP06 model equations (see Methods).

When, as shown in Fig 12, the simulation was run again (CL* = 350 ms, ω = 2.4, *clv* = 35 ms) after removing the time-dependency of I_CaL_ (see Methods) at the time of the switching, the ability of the dynamic pacing to stop alternans was lost with the Shift_CL_ of the corresponding space of states (see Fig 7C). Such ability was preserved when the same intervention was performed on I_Kr_ and I_Ks_. Thus, given a certain range of beat-to-beat CL variability, a mechanism emerges that counteracts other instability sources. This can be unmasked by dER_CL_ but not by dER_DI_ representations, appears to be linked to the time-dependence of I_CaL_, and can make repolarization more stable.

## Discussion

Measurement of the electrical restitution of cardiac ventricular AP has been used previously to study memory and stability of repolarization, which have also been related to hysteresis in APD vs DI that develop in dynamic conditions [25, 29, 43]. By means of simulations carried out on a human ventricular AP model [34], we show that hysteresis affects both APD vs DI and APD vs CL representations of dER, and that the latter is a better marker of memory and stability. We present a generalization of the mechanism underlying hysteresis in different pacing conditions, investigate the role of 3 plateau ion currents in modulating this property, and analyze the link between hysteresis and AP stability after transient perturbations of beating rate. We have found that, at high pacing rate, by increasing the frequency of periodic beat-to-beat CL changes, the space of dER_CL_ states changes from the typical unimodal curve to an hysteresis loop; dER_DI_ does the same, though for much slower beat-to-beat CL changes and to a smaller extent. A randomly varying pacing frequency leads to a scattered dER_CL_ space of states covering an area whose shape and size depend on the pacing parameters. The geometry of the space of states for a given pacing protocol is mainly determined, in the AP model under study, by the interplay of voltage and time-dependency of I_CaL_ and I_Ks_. At high pacing frequency, it is the size of the dER_CL_ space (and not that of the dER_DI_) that correlates with repolarization stability after a single missing beat, while periodic more than random pacing is effective in damping sudden APD oscillations.

### ER_CL_ vs ER_DI_

APD vs DI representations (dER_DI_) of sequences of cardiac APs have long been adopted for measuring dynamic electrical restitution [44], as opposed to APD vs CL (dER_CL_). The main reason for this choice is the meaning of the ER_DI_ slope for AP dynamics under constant pacing CL, with a slope > 1 amplifying, and < 1 suppressing APD oscillations after CL disturbances [45], even though the general applicability of such a concept remains controversial [3, 14, 34, 46]. Moreover, the concept holds only as long as CL is constant, whereas most of the time we are interested in dynamic conditions where CL is the independent variable driving both APD and DI. Wu and Patwardhan developed an experimental protocol to make DI the independent variable [25] by timing each consecutive current stimulus with a programmed DI, which can follow any time-law, and is therefore independent from the preceding APD. More recently, Elizabeth Cherry and coworkers from one side and Sharon Zlochiver and coworkers from another applied a constant-DI pacing protocol, which appears to be effective in differentiating and controlling alternans mechanisms [47, 48]. However, time-clamping DI can hardly be associated with any *in vivo* condition and, if this does not make it less appropriate for investigating membrane properties, it does make this approach less clear from a practical point of view. In addition, the hysteresis in dER_DI_ representations is less prominent in the TP06 model when compared to that in dER_CL_. Finally, the conditions that bring about hysteresis in dER_DI_, which have been described earlier [25], are different from those in dER_CL_ (Fig 5), and, since in our simulations mainly the latter seem to be critical for AP stability, we are devoting most of our work to exploring AP sequences as dER_CL_.

### Hysteresis and memory

The use of restitution hysteresis as a marker and measure of short-term AP memory has been studied elsewhere for dER_DI_ [15, 25, 26, 29, 49] and several models have been proposed to investigate it at different CL values [21, 43, 32]. Short-term AP memory has been seen as a function that accumulates information during systole and dissipates it during diastole [14]. Our own simulations confirm that the information concerning the recent past and future of an AP can be measured by the hysteresis of dER, but they also suggest that such information is shared in a diverse way among systole (APD) and diastole (DI) in different dynamic pacing conditions, with a prevalence of the former in the model under study. Others have found, by looking at dER_DI_ hysteresis, much larger Shift_DI_ values at comparable pacing rates in porcine ventricular tissue [26] and it is likely that a different balance between Shift_DI_ and Shift_CL_ would be found in different AP models and in different species. In fact, heterogeneity of restitution properties has been documented even in different cell types from the same heart [49], and the two components of the delayed rectifier potassium current, differently contributing to dER, have also been shown to be heterogeneously distributed along the base-apex direction [50].

If we pace the TP06 AP model with a periodically changing CL* centered at 350 ms within a ± 20% variability range, and regardless of whether we drive CL or DI to achieve such changes (not shown), we observe measurable hysteresis in dER_CL_ for ω values greater than 0.2, whereas in dER_DI_ hysteresis is only measurable for ω values lower than 1.0, with a small ω interval where both are present (Fig 5C). Hysteresis in dER_CL_ and not in dER_DI_ means that information is stored in APD, since at any CL (n^th^ beat), the APD choice for the “future” (n+1^th^ beat) does not depend on the previous DI (no choice, monotonic dER curve) but on the previous CL (double choice depending on previous history, i.e. hysteresis), and therefore on APD (= CL—DI). The information concerning the recent pacing history is stored in CL and not in DI, thus we can conceive it as accumulated in APD (see for example Fig 4C left, hysteresis in dER_CL_ and not in dER_DI_). Previous DI does not distinguish whether CL is increasing or decreasing whereas previous APD (and therefore CL) does. In other words, at high pacing frequency (CL* < 400 ms), if CL changes periodically from beat-to-beat at a fast rate (ω > 1, see Fig 5C), AP memory accumulates mainly on APD. For slower CL changes, the opposite happens, since what it is that discriminates between the two possible future states of the system now depends on DI (whether it increases or decreases) and, in fact, can be seen in dER_DI_ (Fig 4C right). Thus, to characterize short-term AP memory, both representations should be explored, since each contains information that cannot be derived from the other, and this suggests that short-term AP memory is shared between systole (APD) and diastole (DI) depending on pacing conditions.

### Pacing perturbations

Although the link between hysteresis and repolarization stability has been previously described in the case of dER_DI_ [25], our results show that dER_CL_ can be used to understand and predict, for instance, conditions when otherwise silent pathologies of ion currents might become pro-arrhythmic, and also how pacing rate variability can control the transition to arrhythmias. An example of this can be found, for instance, in the defective I_Ks_ due to KCNQ1 mutation of Type 1 long QT syndrome [51], which we simulated in the experiment in Figs 11 and 12. The inherited APD prolongation observed in this pathology becomes malignant under adrenergic stimulation when, it has been proposed [52], part of the repolarization reserve (mainly I_Kr_) is rate-dependently removed, and a pacing perturbation can initiate alternans [53] and, in turn, trigger arrhythmias. In these conditions, however, pacing variability introduces a protecting mechanism from arrhythmias, which can be monitored in the shape of the corresponding space of dER_CL_ states (Fig 7B). In fact, although under these circumstances (periodic pacing, CL* = 350 ms, *ɷ* = 2.4, *clv* = 35 ms, see Fig 11A) the increase in dER_DI_ slope (Fig 9B) would predict a decreased repolarization stability, the large beat-to-beat oscillations of CL bring about an increase in Shift_CL_ and, with this, in APD stability too (Figs 10C and 11A). Accordingly, for values of ω = 2 (Shift_CL_ diminished by 40%, Shift_DI_ unmodified) and lower (see Fig 11C), the switch from constant to periodic pacing did not stop alternans. The 68% increase in the slope of classical ER_DI_ (Fig 9A) does predict an increased instability under G_Ks_ decrease, but is measured under constant pacing.

The ability of periodic pacing to prevent alternans is better described by dER, which captures electrical restitution properties under dynamic pacing, like that shown in Fig 8C and 8D (middle panels), and dER_CL_ rather than dER_DI_, with its hysteresis behavior, provides a link between this ability and short term AP memory.

### Role of active membrane properties

Ion currents dynamics is known to determine and affect cardiac electrical restitution [26, 54], hence our interest in studying its role in shaping the space of states in general, and particularly that of dER_CL_. The difficulty with the role of repolarization ion currents and ER is the fact that their up/down-regulation brings about APD changes which, per se, can affect restitution properties [55]. We therefore consider changes in the maximum conductance of different ion currents that lead to the same shortening/prolonging effect on APD (Fig 7A and 7B). Taken together, our data suggest a major involvement of both the amplitude and time-dependency of I_CaL_ in determining size, thickness and slope of the hysteresis loop in dER_CL_. Accordingly, when G_CaL_ is increased/decreased, Shift_CL_ also increases/decreases, and when the time-dependence of I_CaL_ is removed, Shift_CL_ is virtually abolished. This latter finding is particularly interesting when compared with the results on rabbit ventricular myocytes of Mahajan and coauthors [56], who proposed that flattening ER_DI_ by inhibition of I_CaL_ inactivation rather than by current block could be viewed as a way to improve repolarization stability without depressing contractility. Thus, our own results also confirm that targeting kinetics of I_CaL_ recovery rather than its amplitude may be a promising anti-arrhythmic strategy.

The repolarizing I_Ks_ partially counterbalance I_CaL_ all over the AP and particularly in the late repolarization phase. Thus, when its G_max_ is reduced or the time-dependence of I_Ks_ is removed, the positive effect of I_CaL_ on Shift_CL_, partially freed from this restraint, can develop even further (Fig 7C, central column). The fast kinetics of I_Kr_ (compared to that of I_Ks_) perhaps explain why removal of time-dependence from this current does not modify Shift_CL_.

By demonstrating the same effect with a verapamil-induced reduction of I_CaL_ in isolated ventricular tissue from pigs, Guzman [26] noted the contrasting effect of slope and memory on stability, suggesting a careful consideration of both effects when testing antiarrhythmic properties of I_CaL_ blockers. We have extended this notion to our data on dER_CL_ and note the fact that in the simulations in Fig 7B, the corresponding dER_DI_ curves are all unimodal (not shown), and only much smaller ω (~ 0.2) values made them assume hysteresis behavior. Thus, both phenomena pertain to AP memory, though only the one at higher ω values (dER_CL_), as pointed out above, can be extrapolated to comparable random pacing conditions, and explain the protective effect on repolarization after pacing perturbations.

## Limitations

The present work has been performed on a specific model of human ventricular AP. Although preliminary results obtained with additional models (2 humans and 1 rabbit), which we briefly mention above, seem to confirm our conclusions, only further work could completely rule out a possible model-dependency in our findings. This is particularly true for our discussion about the role of inactivation of I_CaL_ in modulating memory and stability, where models with a more detailed description of calcium current kinetics and intracellular calcium handling could better prove our conclusions.

## Conclusions

Our data show that, at high pacing frequency, information concerning the recent pacing history is stored differentially in systole (APD) and diastole (DI). This kind of AP memory, particularly that measured in dER_CL_, turns on in extreme pacing variability conditions, where it counterbalance the pro-arrhythmic effect of abrupt changes in CL. Any partial or complete loss of this safety mechanism, as in the case of changes in amplitude or kinetics of repolarization ion currents, exposes ventricular repolarization to an increased risk of arrhythmias, which can be monitored in dER_CL_ and not in dER_DI_. Screening ventricular AP repolarization for APD and DI memory, both *in vivo* and in the growing family of cardiac human ventricular AP models, may lead to a refinement of clinical criteria for electrical instability and may benefit many aspects of cardiac physio-pathology, such as an understanding of the latent instability of certain inherited repolarization pathologies, the design of new antiarrhythmic drugs, or the challenge of intelligent sensing in artificial pacemakers. Of further relevance in this regard is our finding of the greater effectiveness of periodic rather than random pacing in dampening sudden APD oscillations, for which we have provided a statistical explanation (see S1 and S2 Figs), and which again is more effectively revealed in the dER_CL_, rather than dER_DI_ representations. One possible application of this finding might be in designing pacing protocols for artificial pacemakers, where a predefined periodic variability can be added to the pacing rate to reduce susceptibility to arrhythmias.

## Supporting information

S1 Fig APD displacement after a missing beat. This figure shows how the data in Fig 8B were obtained. AP sequences were simulated under constant, periodic, and random pacing conditions (CL* = 350 ms, ω = 2.4, clv = 35 ms), where a given n^th^ beat was missing and the difference between the 50 APDs following the missing beat and the those of the unperturbed sequence was measured. The same was done for sequences where the missing beat was the (n+1)^th^, and then (n+2)^th^, until (n+19)^th^. Panel a reports the averages, for each pacing conditions, of the 20 traces described above. The same was done for AP sequences where APD was 12.5% prolonged by modifying maximum conductance of ion channels (see Results).
(DOCX)

S2 Fig CL distribution in random and periodic pacing. (A) A periodically changing (CL* = 350 ms, ω = 2.4, clv = 35 ms) CL sequence is reported on the left and a randomly changing (CL* = 350 ms, clv = 35 ms) on the right. (B) The absolute values of beat-to-beat CL changes are reported in the two instances. The horizontal broken line denotes a given threshold of 30 ms. (C) Corresponding normalized frequency distributions. The number of events with abs(ΔCL) > 30 ms are measured by the red areas A_p_ (periodic pacing) and A_r_ (random pacing). (D) The ratio between A_p_ and A_r_ is reported versus any given threshold of abs(ΔCL).
(DOCX)

S3 Fig Time-independent version of three ion currents. The figure shows at the top a simulated AP waveform at CL = 350 ms and, below, current traces in their native form (red), and derived with the fitting procedure (blue).
(DOCX)

## Abbreviations

AP: action potential
APD: action potential duration at V_m_ = -60 ms
CL: pacing cycle length
CL*: average value of conditioning pacing CL
*clv*: half range of pacing variability around CL*
DI: diastolic interval
ER: classic electrical restitution
dER: dynamic electrical restitution
ER_CL_: electrical restitution curve measured as APD vs CL
ER_DI_: electrical restitution curve measured as APD vs DI
N_b_: number of beats
ω: rate of CL changes
Shift_CL_: central thickness of dER_CL_ hysteresis
Shift_DI_: central thickness of dER_DI_ hysteresis
RD: rate dependence
TP06: Ten Tusscher et al. 2006 human ventricular AP model [34]
V_m_: membrane potential
