# Supplementary material for: Short-term action potential memory and electrical restitution: A cellular computational study on the stability of cardiac repolarization under dynamic pacing
Source: PLoS One. 2018 Mar 1;13(3):e0193416. doi: 10.1371/journal.pone.0193416 (PMC5832261; doi:10.1371/journal.pone.0193416)

**S1: Appendix**

*APD distribution during random and periodic pacing.*

Fig 8B shows that, at high pacing rate, the effect of a single missing beat on succeeding APDs lasts for a shorter length of time if pacing rate is constant, longer if the pacing rate is periodic and even longer if it is random (periodic and random pacing within the same *clv*, and with the same maximum value of beat-to-beat ΔCL). The same is true of APD-prolonged conditions except for the cases, G_CaL_ up-regulation and G_Ks_ down-regulation under constant pacing, where perturbation triggers alternans. The data in Fig 8B were obtained as follows: ΔAPD (absolute value of the difference between perturbed and non perturbed APD) was measured as a function of the number of beats after the missing one, in the 3 pacing conditions (CL^*^ = 350 ms, *clv* = 35 ms, ω = 2.4). Ten such ΔAPD sequences were obtained for each pacing condition by repeating the measure for as many positions of the missing beat and averaging them in order to maximize the effect of the type of pacing and minimize any small beat-to-beat differences. The results with this protocol are reported here; the horizontal line shows the arbitrary threshold value of 2 ms chosen for ΔAPD in order to approximate recovery of the unperturbed APD.


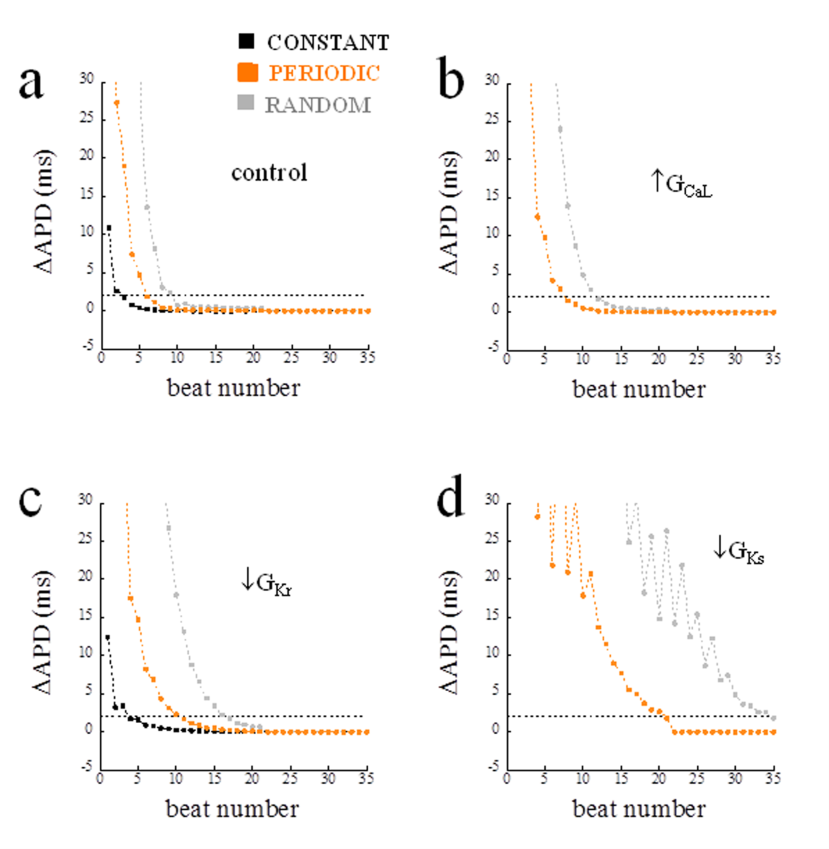

Supplement: S1 Fig — This figure shows how the data in Fig 8B were obtained. AP sequences were simulated under constant, periodic, and random pacing conditions (CL* = 350 ms, ω = 2.4, clv = 35 ms), where a given nth beat was missing and the difference between the 50 APDs following the missing beat and the those of the unperturbed sequence was measured. The same was done for sequences where the missing beat was the (n+1)th, and then (n+2)th, until (n+19)th. Panel a reports the averages, for each pacing conditions, of the 20 traces described above. The same was done for AP sequences where APD was 12.5% prolonged by modifying maximum conductance of ion channels (see Results). (DOCX) [file pone.0193416.s001.docx]
