# Supplementary material for: Short-term action potential memory and electrical restitution: A cellular computational study on the stability of cardiac repolarization under dynamic pacing
Source: PLoS One. 2018 Mar 1;13(3):e0193416. doi: 10.1371/journal.pone.0193416 (PMC5832261; doi:10.1371/journal.pone.0193416)

**S2: Appendix**

*APD distribution during random and periodic pacing.*

The time course of CL and abs(ΔCL) are reported in panels A and B of the figure for periodic and random pacing. The ɷ value in Equation 3 was chosen in order for the beat-to-beat ΔCL values to cover the same range as those in random pacing. We have shown that, at high pacing rate, it is the size of beat-to-beat ∆CL that determines the vertical width of the space of states which, in turn, stabilizes AP repolarization. If we hypothesize that only beats with ∆CL above a certain threshold (vertical broken line in panel C) play such a role, we find that the total number of such beats (area Ap in panel C) is always greater in periodic than in random pacing (area A_r_), independently of the chosen threshold. Their ratio, in the case for example of a threshold of 20 ms, is about 1.5, which can explain the control results in the histogram of Fig 8, where Nb goes from 6 (periodic pacing) to 9.23 (random pacing), therefore making periodic pacing 50% more effective in restoring the unperturbed AP waveform after the missing beat.


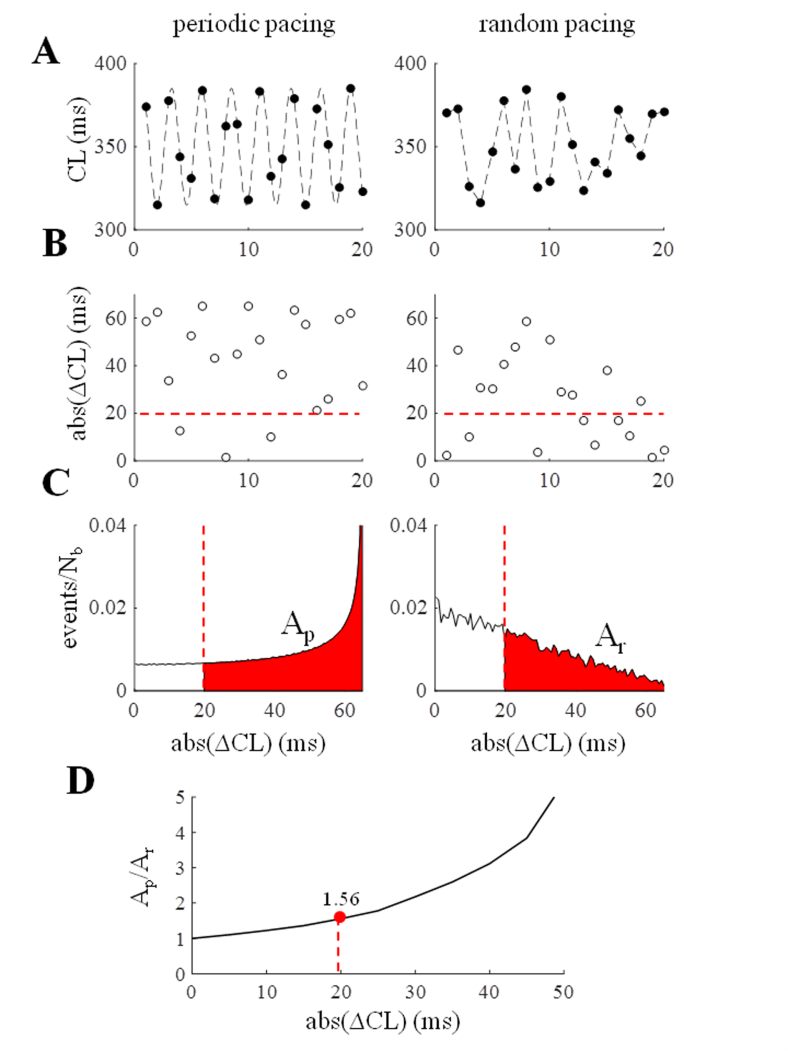

Supplement: S2 Fig — (A) A periodically changing (CL* = 350 ms, ω = 2.4, clv = 35 ms) CL sequence is reported on the left and a randomly changing (CL* = 350 ms, clv = 35 ms) on the right. (B) The absolute values of beat-to-beat CL changes are reported in the two instances. The horizontal broken line denotes a given threshold of 30 ms. (C) Corresponding normalized frequency distributions. The number of events with abs(ΔCL) > 30 ms are measured by the red areas Ap (periodic pacing) and Ar (random pacing). (D) The ratio between Ap and Ar is reported versus any given threshold of abs(ΔCL). (DOCX) [file pone.0193416.s002.docx]
