# Supplementary material for: Short-term action potential memory and electrical restitution: A cellular computational study on the stability of cardiac repolarization under dynamic pacing
Source: PLoS One. 2018 Mar 1;13(3):e0193416. doi: 10.1371/journal.pone.0193416 (PMC5832261; doi:10.1371/journal.pone.0193416)
Supplement: S3 Fig — The figure shows at the top a simulated AP waveform at CL = 350 ms and, below, current traces in their native form (red), and derived with the fitting procedure (blue). (DOCX) [file pone.0193416.s003.docx]

**S3: Time-independent version of three ion currents.**


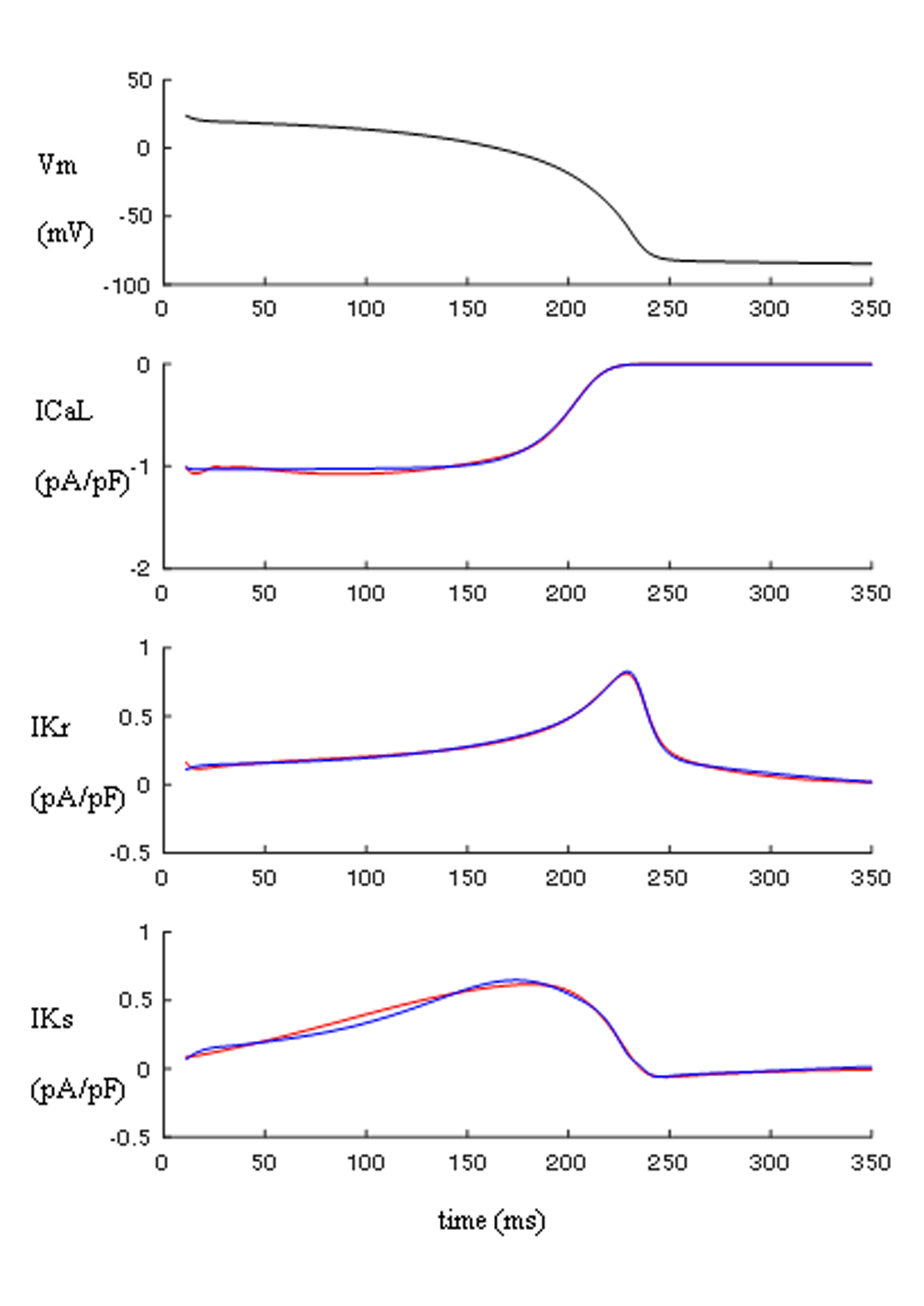
The I-V relationship of each current was obtained by recording it during the AP paced at constant CL=350 ms, fitting the I-V curve with 9^th^ order polynomials (I=mo+m1*Vm+m2*Vm^2^+ …, fitting coefficients reported below for each ion current) and simply implement ion currents during the AP according to the fitted values, instead of solving the corresponding gating equations. The replacement concerned all AP trajectory except for the first 9 ms after AP onset, where polynomial fits did not reproduce the three ion currents correctly. This is an acceptable approximation in our study, which is mainly concerned with repolarization dynamics and thus on the later phases of the three currents.

**I_CaL_**

m0=-0.94024;

m1=-0.01447;

m2=0.00082343;

m3=-3.0635e-06;

m4=-1.015e-06;

m5=-3.3756e-09;

m6=6.7127e-10;

m7=1.4874e-11;

m8=1.2663e-13;

m9=3.9514e-16;

**I_Kr_**

m0=0.31617;

m1=-0.0088694;

m2=-1.0741e-6;

m3=-6.1713e-7;

m4=2.6316e-8;

m5=6.3622e-10;

m6=0;

m7=0;

m8=0;

m9=0;

**I_Ks_**

m0=0.63472;

m1=-0.0070589;

m2=-0.0010982;

m3=-1.88e-05;

m4=9.0419e-07;

m5=3.4223e-08;

m6=1.5124e-11;

m7=-1.281e-11;

m8=-1.8176e-13;

m9=-7.7175e-16;
